# Supplementary material for: A time-resolved picture of our Milky Way’s early formation history
Source: Nature. 2022 Mar 23;603(7902):599–603. doi: 10.1038/s41586-022-04496-5 (PMC8942851; doi:10.1038/s41586-022-04496-5)
Supplement: Supplementary file 1 — Supplementary Information sections 1. The data; 2. The sample’s selection function; 3. The intrinsic age scatter of the thick disk; 4. The old disk stars ‘splashed’ by the merger with the Gaia-Sausage-Enceladus satellite galaxy; 5. Comparison of the age–metallicity relation with the literature. [file 41586_2022_4496_MOESM1_ESM.pdf]

nature portfolio

https://doi.org/10.1038/s41586-022-04496-5

Supplementary information

A time-resolved picture of our Milky Way's early formation history

In the format provided by the authors and unedited

Supplementary text

1. The Data

Determining spectroscopic absolute magnitudes, M_K **:** To estimate the age of a subgiant star precisely, one needs to determine its luminosity (or absolute magnitude) precisely. This can be based on parallax estimates from Gaia for stars with distances of ≤ 2 kpc. Beyond 2 kpc, parallax uncertainties become larger than 10% and spectroscopic constraints on the absolute magnitudes are necessary to ensure sufficient age precision. In principle, on can use spectroscopic estimates of a star's surface gravity, $\log g$, to estimate its luminosity. However, in practice, $\log g$ estimates have non-negligible systematic errors, especially severe for metal-poor stars, which are possibly due to calibration errors.

To get unbiased spectroscopic luminosity estimates, we derive M_K , the absolute magnitude in the 2MASS K band, from LAMOST spectra using a data-driven neural network model, similar to [1], where it was applied to OB stars. Here, our neural network contains 5 layers, with 512, 256, 128, 64, and 32 neurons, respectively. We adopted the rectified linear activation function (ReLU) and carried out the training with the *Pytorch* framework. Stars with precise Gaia parallaxes are adopted as the training set, where (geometric) M_K can be derived from the 2MASS apparent magnitudes [2] and the Gaia distance from [3]. Binary stars are discarded from the training set by iteration.

Extended Figure 3 illustrates the validation of these spectroscopic M_K estimates, using test sets of stars that either have precise geometric M_K or repeat measurements from the LAMOST duplicate spectra. These test sets verify the quality of the spectroscopic M_K estimates, except for unresolved binary or multiple stars, for which the geometric M_K are systematically brighter than the spectroscopic M_K estimates. For such binaries, the geometrically determined M_K are brighter due to the contribution of the secondary (up to 0.75 mag in the equal-mass binary). Interestingly, the spectroscopic M_K estimates seem to be good approximations of the primary components true M_K . This is apparent in the case of equal-mass binaries: the spectra of the two components are identical, and the normalized spectra yield the true M_K of either component. Actually, this serves as an efficient way of identifying large samples of unresolved binaries and multiple stars from the LAMOST survey spectra and Gaia parallax [1, 4]. Repeat measurements illustrate that our method is able to deliver spectroscopic M_K estimates precise to better than 0.1 mag from high-S/N ($S/N > 80$) LAMOST spectra for metal-rich ([Fe/H] $\gtrsim 0$) stars, which yields a distance precision of ∼3%. The M_K precision decreases with both S/N and metallicity. For example, for metal-poor stars of [Fe/H] $\simeq -1$, the spectroscopic M_K has a precision decreases from 0.5 mag at S/N = 20 to 0.15 mag at high S/N (> 80). We assign an error to the spectroscopic M_K estimate for each subgiant star according their S/N and [Fe/H], based on dispersion of the repeated measurements.

Determining stellar ages : All stellar ages are estimated by combining spectroscopic, photometric and astrometric observables x with stellar isochrones in a Bayesian framework. The posterior probability distribution of any target parameters θ, $P(\theta|x)$ – describing the star's physical properties, including age – is then given by the product of the likelihood $P(x|\theta)$ and the prior probability distribution $P(\theta)$,

$$P(\theta|x) \propto P(x|\theta) \times P(\theta). \tag{1}$$

Here, the observables x are the (Galactic) sky coordinates l, b; the parallax ϖ from Gaia eDR3 with the zero-point offset correction from [5]; the spectroscopic stellar labels $T_{\rm eff}$, [Fe/H], [α/Fe]; the spectroscopic absolute magnitude M_K^{spec} from the LAMOST spectra; and a set of multi-band apparent magnitudes: G, BP, RP from Gaia eDR3 [6] and J, H, $K_{\rm s}$ from 2MASS [2]. The physical parameters (θ) characterizing the star and predicting the observables x are age (τ), mass M, initial composition $[\mathrm{Fe/H}]_{ini}$, and distance d.

We now denote the spectroscopic labels among the observable x, ($T_{\rm eff}$, [Fe/H], [α/Fe], M_K^{spec}) as s, and the observed apparent magnitudes (G, BP, RP J, H, $K_{\rm s}$) as β, respectively. Analogously, we denote the spectroscopic labels and absolute magnitudes predicted by the stellar isochrones for a star with given θ as s' and β', respectively. With this notation, the likelihood function $P(x|\theta)$ can be expressed as

$$P(\varpi, s, \beta|d, \tau, M, [\mathrm{X/H}]_{\rm ini}) = \tag{2}$$
$$P(\varpi, s, \beta|d, s', \beta')\ P(s'|\tau, M, [\mathrm{X/H}]_{\rm ini})\ P(\beta'|\tau, M, [\mathrm{X/H}]_{\rm ini}) \tag{3}$$

$$P(\varpi, s, \beta|d, s', \beta') = P(\varpi|d)\ P(s|s')\ P(\beta|d, \beta', A) \tag{4}$$

$$P(\varpi|d) := \mathcal{G}(\varpi - 1/d, \sigma_\varpi), \tag{5}$$
$$P(s|s') := \mathcal{G}(s - s', \sigma_s), \tag{6}$$
$$P(\beta|d, \beta', A) := \mathcal{G}\Big(\beta - \big(\beta' + (5\log d - 5) + A\big), \sigma_\beta\Big), \tag{7}$$

Here [X/H] denotes both [Fe/H] and [α/Fe], $\mathcal{G}$ the Gauss function, and σ the measurement uncertainties of the observables. We also need to know the extinction A to a star's distance d to link its absolute magnitude to its apparent magnitude. We have determined the most likely extinction for each star (see below) and keep it fixed in the age determination. The total likelihood of the data set then results from the product of the the stars' individual likelihoods:

$$P(s|s') = \Pi_i\, p(s_i|s_i') \ \text{ and } \ P(\beta|\beta') = \Pi_i\, p(\beta_i|\beta_i'). \tag{8}$$

We adopt the Yonsei-Yale (YY) isochrones [7] for our analysis. For YY isochrones, the atomic diffusion of heavy elements is not modelled, which implies that $[\mathrm{Fe/H}]_{\rm ini}$ is identical to $[\mathrm{Fe/H}]_{\rm now}$. While this might cause some uncertainty for main-sequence turn-off stars [8], atomic diffusion is negligible for the subgiant stars considered here. We interpolate the original public YY

isochrones [7] to a fine grid with a step of 0.05 dex in [Fe/H], and 0.2 Gyr in age. The YY isochrones adopt as "solar metallicity" the abundances of [9], the LAMOST spectroscopic analysis for [Fe/H] adopts those of [10]. Therefore we modify the LAMOST [Fe/H] by adding −0.12 dex to the DD-Payne estimates to match the solar abundance scale of the YY isochrones for age determination.

The YY isochrones provide photometry predictions for the UVBRI and ESO JHK passbands, but not for the Gaia passbands. We convert the UVBRI photometry predictions to the Gaia passbands using the relation of [6]. Similarly, we convert the absolute magnitudes in the ESO JHK passbands predicted by the YY isochrones to the 2MASS JHKs passbands using the relation of [11].

For estimating the parameters θ, we need to spell out a prior probability distribution function $P(\theta)$. For [Fe/H], we adopt a flat distribution. For the priors on age and distance distribution, we adopt broad priors that depend on the sky position (l, b). These are taken from the measured 3D stellar mass distribution for mono-age populations of [12]. Given the expected quality of our age and distance estimates, these priors are not unduly constraining our estimates. Finally, assign a weight to each isochrone grid point to account for the underlying stellar mass distribution along the isochrones, based on a Kroupa IMF [13] .

Given the posterior probability distribution function $P(\theta|x)$ of a star, we take the weighted mean of the posterior $(\bar{\tau})$ as the age estimate of the star, and the standard deviation (σ_τ) as an estimate of the uncertainty:

$$\bar{\tau} = \frac{\Sigma w_i \tau_i}{\Sigma w_i}, \tag{9}$$

$$\sigma_\tau = \sqrt{\frac{\Sigma w_i}{(\Sigma w_i)^2 - \Sigma w_i^2} \Sigma w_i (\tau_i - \bar{\tau})^2}. \tag{10}$$

Here, the weight w is the posterior probability distribution $P(\theta|x)$, and the summation is done over all grids of θ (τ, M, $[\mathrm{Fe/H}]_{ini}$, d) selected for the age determination using a box cut in T_{eff} , M_K , and [Fe/H] , and d. The box size in each dimension is adopted as the 3σ value of the parameter uncertainty, but an lower and upper limit are set for the box size in T_{eff} , M_K , and [Fe/H] , which are respectively 200 K and $0.3\times T_{\mathrm{eff}}$ in T_{eff} , 0.3 and 2.0 mag in M_K , and 0.2 and 4.0 dex in [Fe/H] .

Determinig Dust Extinction: Estimates of the dust extinction towards individual stars are required for estimating the ages and distances (see Equations 7 and 11). To obtain the extinction A for individual stars, we determine the reddening $E(B-V)$ and total-to-selective extinction coefficients R, so that $A = R \times E(B-V)$. The $E(B-V)$ for individual stars can derived by comparing their spectroscopically predicted intrinsic colors with the observed colors, similar to the method described in [1]. Specifically, the intrinsic colors are derived from the spectroscopic stellar parameters with the so-called star-pair method: we assume stars with the same stellar parameters have the same intrinsic colors [14], and regress the intrinsic colors using the dereddened colors

of a set of control stars that have known (and small) extinction at high Galactic latitudes, based on the SFD map [15] that has been corrected for a 14% overestimate [16]. Typical uncertainty of the reddening $E(B-V)$ estimates is 0.01-0.02 mag.

We have derived the total-to-selective extinction coefficients R in the various individual passbands by convolving Kurucz model spectra [17] that have the same stellar parameters as the sample stars with the extinction curves from [18]. The derivation of total-to-selective extinction coefficient R does not only depend on the stellar SED, i.e., stellar parameters, but also depend on the $E(B-V)$ itself. We adopt $E(B-V)$ from the 3D extinction map of [19] as an initial estimate to derive the R. Subsequent iteration might give a more accurate determination of R; but we found that small changes in R do not change our final results significantly, considering the relatively small total-to-selective extinction coefficients in the majority of the passbands, for example, $R_{Ks} \sim 0.34$, $R_J \sim 0.56$, $R_J \sim 0.72$, $R_G \sim 2.5$.

Determining the Stars' Orbital Actions: We combine the Gaia astrometric parallax and LAMOST spectroscopic M_K to estimate the distances to our sample stars. Specifically, we use

$$\log d = \frac{w_1 \times \log d_{geom} + w_2 \times \log d_{spec}}{w_1 + w_2}, \tag{11}$$

$$\log d_{geom} = 3 - \log(\varpi - \varpi_0), \tag{12}$$

$$\log d_{spec} = 0.2(Ks - \mathrm{M}_K{}^{spec} - A_{Ks} + 5), \tag{13}$$

where ϖ_0 is zero-point of the Gaia DR3 parallax generated with the method of [5], A_{Ks} the extinction in Ks band. The weights w_1 and w_2 are the inverse variances of $\log d_{geom}$ and $\log d_{spec}$, respectively.

We then compute the orbital actions and angles of our sample stars with the *Galpy* by [20], based on the LAMOST radial velocity and Gaia proper motion. It is well known that the LAMOST radial velocity estimates exhibit a systematic zero-point offset by a few km/s [21, 22], which we correct by adding 4.9 km/s, a value derived from the comparison with the Gaia radial velocity measurements for bright stars. For applying the *Galpy*, we adopt the *MWPotential*2014 potential model, which combines a power-law model with cut-off for the MW bulge, a Miyamoto–Nagai model for the disk, and a NFW model for the halo (see details in [20]). We assume the Sun is located at $R_\odot = 8.178$ kpc, as suggested by [23], and $Z_\odot = 10$ pc above the disk mid-plane [12]. We assume LSR = 220 km/s, and the solar motion with respect to the LSR, $(U_\odot, V_\odot, W_\odot) = (-7.01$ km/s, 10.13 km/s, 4.95 km/s$)$ [24]. We use the *Orbit* module in the *Galpy* to integrate the orbits of our subgiant sample stars to their ages, and use the Staeckel approximation to calculate the actions, angle and frequency of the stars' orbits. For the analysis in this present paper, we only use the angular momenta, J_ϕ.

2. The Sample's Selection Function

For our analysis we need to understand how the chances of a star to be in the sample, which depends on its position in the Galaxy (or distance from the Sun), as well as its age, stellar mass and [Fe/H] , i.e. the sample's selection function (see e.g. [25]).

The age-[Fe/H] relation from Gaia mock catalog To assess the impact of possible selection effects, we apply our target selection to the Gaia mock catalog of [26], investigating its impact on the age–[Fe/H] relation. We generate the Gaia mock stars in the same footprint as the LAMOST **DR5**, and with the same magnitude range in each sky direction as the LAMOST stars. We then apply the same criteria in the $T_{\rm eff}$ -$\rm M_{\it K}$ plane as for our LAMOST sample for selecting subgiant stars. As Ks-band magnitudes are not provided in the Gaia mock catalog of [26], we predict $\rm M_{\it K}$ from the parameters $T_{\rm eff}$, $\log g$, [Fe/H] and age provided there. We do that with a neural network model trained on the PARSEC isochrones [27] that had been used to produce the Gaia mock catalog. The Gaia mock catalog assigns a constant age of $\tau = 11\,$Gyr for the thick disk, and a constant age of $\tau = 13\,$Gyr for the halo. Here we assign a random error of 10% to the ages of all the mock stars.

The resulting age-[Fe/H] distribution of the Gaia mock sample (1,250,000 stars) is shown in **Extended Figure 5**. The left panel of **Extended Figure 5** presents the age-[Fe/H] distribution, which shows a continuous age distribution, rather than separated sequences as seen in the observations. This implies that the structures in the age-[Fe/H] distribution that we found in the actual data are unlikely to be consequences of selection effects. Note that at the metal-rich end of the age range of 2–3.5 Gyr, there is a turn-off feature for the $p(\tau|\text{[Fe/H]})$. This is due to a sudden change of [Fe/H] dispersion assigned in Gaia mock catalog at 3 Gyr: at the younger ages, the [Fe/H] dispersion is smaller so that there are more metal-rich stars of [Fe/H] > 0.3 for $\tau > 3\,$Gyr.

Due to the finite number of fibers, LAMOST has subsampled targets in the color-magnitude diagram (CMD), rather than observing all stars within the magnitude limit [14]. It is difficult to apply the exact LAMOST target selection in the HR diagram to our mock sample as the latter is complex, which incorporates the fiber allocations [14]. However, because the survey selects stars in the full CMD homogeneously and randomly, the bias in the resultant [Fe/H] distribution (and conjecturally, age distribution) should be negligible [21, 28, 29]. To mimic this selection process, we randomly select a subset of the Gaia mock subgiants to match the star number of the LAMOST sample. The resultant age–[Fe/H] relation is shown in the right panel of **Extended Figure 5**. While the figure presents some artifacts at the younger age side of $\tau < 9\,$Gyr due to the small number of stars, the overall conclusion remains unchanged.

Accounting for the age-dependent effective survey volume: Fig. 3 of the main text shows the age distribution of the $\alpha - enhanced$, early

thick disk, reflecting its star-formation history. However, the observed age–[Fe/H] distribution of subgiant stars is related to the *true* star-formation history by a number of factors:

$$N_{\rm obs}^{\rm subgiant}(\tau, [{\rm Fe/H}]\,) = \tag{14}$$

$$\int\int\int\int \rho(l,b,r) f(\tau, [{\rm Fe/H}]\,|l,b,r)\, S_{\vec{x}}(l,b,r)\, S_{mass}(m|\tau, [{\rm Fe/H}]\,) dl db dr dm, \tag{15}$$

where r is the distance, m the stellar mass, $\rho(l,b,r)$ the underlying stellar mass density distribution of our Galaxy in the coordinate system (l,b,r), $f(\tau, [{\rm Fe/H}]\,|l,b,r)$ the normalized underlying distribution of stars in the age and [Fe/H] space, for given (l,b,r). $S_{\vec{x}}(l,b,r)$ is the spatial selection function of the survey and $S_{mass}(m|\tau, [{\rm Fe/H}]\,)$ is the stellar mass function, for which the lower and upper mass boundaries for the subgiant phase are a function of age and [Fe/H] . In contrast, the "true" age distribution or star-formation history, $\Phi(\tau)$, is given by

$$\Phi(\tau) = \int\int\int\int \rho(l,b,r) f(\tau, [{\rm Fe/H}]\,|l,b,r) dl db dr d[{\rm Fe/H}]\,. \tag{16}$$

.

So, $N_{\rm obs}^{\rm subgiant}$ differs from $\Phi(\tau)$ both by spatial selection function $S_{\vec{x}}$ and the selection in stellar mass S_{mass}. These selection effects will not only change the normalization, but may also induce biases in the observed stellar age and [Fe/H] distribution (see e.g. [25]). Therefore we need to model these effects, or correct for them. To account for S_{mass}, we integrate over it to get the fraction of subgiant stars with respect to the full stellar mass function via

$$F_{mass} = \frac{\int_{m_1(\tau,[{\rm Fe/H}]\,)}^{m_2(\tau,[{\rm Fe/H}]\,)} \zeta(m) dm}{\int_{0.08M_\odot}^{100M_\odot} \zeta(m) dm}, \tag{17}$$

where $\zeta(m)$ is the initial mass function of Kroupa (2001) [13], m_1 and m_2 are the lower and upper limit of the mass of subgiant stars, respectively. Their values depend on the age and [Fe/H] , and are derived from the YY isochrones according to our sample selection criteria in the $T_{\rm eff}$ -$\rm M_{\it K}$ plane. For the full stellar mass function, we consider a minimal stellar mass of $0.08\,M_\odot$, and a maximal stellar mass of $100\,M_\odot$.

To account for $S_{\vec{x}}$, we calculate the density-weighted effective survey volume that depends on the geometry and the flux limits of the LAMOST survey, as well as on the luminosity of the objects. We divide the sky into patches of $2^\circ \times 2^\circ$ in (l,b) space. For each patch (or "line of sight"), we can then define

the density-weighted effective volume as

$$V_{\rm eff}(\tau, [{\rm Fe/H}]\,)_{|l,b} = A_{patch} \int_{r_{\rm min}(\tau,[{\rm Fe/H}]\,)}^{r_{\rm max}(\tau,[{\rm Fe/H}]\,)} r^2 \rho(r \mid l, b)\; dr, \tag{18}$$

where A_{patch} is the area of sky patches, $\rho(r)$ the underlying stellar mass density distribution as a function of distance, $r_{\rm min}$ and $r_{\rm max}$ the lower and upper distance boundaries covered by our sample stars, and their values depend on age and [Fe/H] because the intrinsic brightness (absolute magnitude) of the subgiant stars vary with age and [Fe/H] . The distance boundaries are derived using the distance modulus in Gaia G band via

$$\log\, r_{\rm min}(\tau, [{\rm Fe/H}]\,) = (G_{\rm min} - M_G(\tau, [{\rm Fe/H}]\,) - A_{G,r_{\rm min}} + 5)/5, \tag{19}$$

$$\log\, r_{\rm max}(\tau, [{\rm Fe/H}]\,) = (G_{\rm max} - M_G(\tau, [{\rm Fe/H}]\,) - A_{G,r_{\rm max}} + 5)/5, \tag{20}$$

where $G_{\rm min}$ and $G_{\rm max}$ are respectively the bright- and faint-end limited magnitude of the LAMOST survey, determined patches by patches for stars of $S/N > 20$. M_G is the absolute magnitude of the subgiant star, which depends on age and [Fe/H] . Here we adopt M_G rather than $\rm M_K$, because for a given age and [Fe/H] , the M_G of subgiant stars is approximate constant. A_G is the extinction, and it is derived iteratively from a distance – extinction relation constructed for each light of sight making use of the LAMOST stars.

As here we are mostly interested in assessing the observed age distribution of the thick disk, for $\rho(r)$, we only consider the mass distribution of the thick disk. We adopt the thick disk stellar mass density distribution given by Xiang et al. (2018) [12],

$$\rho(R, Z) = \rho_\odot \exp\left(-\frac{R - R_\odot}{H_R}\right) {\rm sech}^{2/n}\left(-\frac{n|Z - Z_\odot|}{2H_Z(1 + \beta(R - R_\odot))}\right)^{1} \tag{21}$$

This equation assumes an exponential decreasing in the radial direction, and a ${\rm sech}^{2/n}$ decreasing in the vertical direction. For an index value $n = 1$, the vertical distribution is isothermal, while it becomes exponential for $n = \infty$. The scale height increases linearly with R to mimic the flaring effect. The parameter values are $\rho_\odot = 0.0037 M_\odot {\rm pc}^{-3}$, $H_R = 1405$pc, $H_Z = 920$pc, $Z_\odot = 114$pc, $\beta = 0.123$, $n = 18.71$ (see Table 3 of [12][2]).

Finally, the underlying stellar distribution in the age-[Fe/H] plane is reconstructed by a simple summation of the individual patches, after correcting for

---

[1] The Equation 14 of [12] is written in the form of $Z - Z_0$ but here we write in the form of $Z - Z_\odot$. This is due to the different definition of Z: [12] place the sun at $Z = 0$ when deriving the values of Z for their sample stars, but here Z is assumed to be the true height above the mid-plane.

[2] Table 3 of [12] given $Z_0 = -114$pc, which means the mass-weighted middle plane of the thick disk is 114 pc below the Sun, i.e., $Z_\odot = 114$pc.

the selection function in spatial volume and stellar mass coverage,

$$N(\tau, \text{[Fe/H]}) = \sum_{i=1}^{N_{patch}} \frac{N_{\text{obs}}^{\text{subgiant}}(\tau, \text{[Fe/H]})_{|l_i,b_i}}{V_{\text{eff}}(\tau, \text{[Fe/H]})_{|l_i,b_i}\, F_{mass}(\tau, \text{[Fe/H]})} \tag{22}$$

The age distribution after correcting for the selection effect is shown in dashed line Fig. 3 in the main text. It illustrates that these selection functions only have a small effect on the *relative* age distribution of the thick disk stars. This is because all member stars are relatively old, and hence differ little in their luminosities.

3. The intrinsic age scatter of the thick disk

As shown in Figs. 2 and 3 in the main text, the old ('thick') disk stars exhibit a tight correlation between age and [Fe/H] extending from [Fe/H] $\lesssim -1$ at 13 Gyr to [Fe/H] $= 0.5$ at 7 Gyr ago. This means that at a fixed [Fe/H] along the sequence, the dispersion of the age distribution is small. Equivalently, at a given age the dispersion of the [Fe/H] is small. Here, we estimate the intrinsic dispersion of age distribution for stars at a given [Fe/H] along the sequence $P(\tau|\text{[Fe/H]})$. We do this rather than estimate $P(\text{[Fe/H]}|\tau)$ because the measurement uncertainties in the age are larger than in [Fe/H]. The scatter in $P(\tau|\text{[Fe/H]})$ taken at face-value is a combined result of the intrinsic dispersion and of age measurement uncertainties. We therefore build a maximum likelihood model of the intrinsic width and estimate the parameters with Markov chain Monte Carlo (MCMC) method.

For simplicity, our model assumes a linear dependence of the mean age ($\bar{\tau}$) on [Fe/H],

$$\bar{\tau}(\text{[Fe/H]}) = \bar{\tau}_0 + a \times \text{[Fe/H]}, \tag{23}$$

where $\bar{\tau}_0$ is the mean stellar age at solar metallicity ([Fe/H] $= 0$). The scatter of the true stellar ages (τ) around this relation follows a Gaussian distribution with possible outliers,

$$P(\tau|\text{[Fe/H]}, \epsilon) = (1-\epsilon)\,\mathcal{G}\Big(\tau - \bar{\tau}(\text{[Fe/H]}), \sigma_\tau\Big) + \epsilon\, R(\tau), \tag{24}$$

where σ_τ is the intrinsic age scatter, which is assumed to be constant for all [Fe/H] values. The ϵ term is a free parameter accounting for possible outliers, for which we adopt a distribution model of

$$R(\tau) = \begin{cases} \frac{1}{\tau_{max} - \tau_{min}}, & \text{for } \tau_{min} \le \tau \le \tau_{max} \\ 0, & \text{else }, \end{cases} \tag{25}$$

where τ_{min} and τ_{max} are the age boundaries of the sample stars.

Given the observation of a star $\{\tau_i,\ \delta\tau_i,\ \text{[Fe/H]}_i\}$, we assume the age measurement uncertainty can be well approximated by Gaussian,

$$P(\tau_i|\tau) = \mathcal{G}(\tau_i - \tau, \delta\tau_i), \tag{26}$$

where $\delta\tau_i$ is the measurement's estimated age uncertainty. Taken together, this yields the likelihood function for a star i of

$$P_i(\tau_i, \delta\tau_i | [\mathrm{Fe/H}]_i, \bar{\tau}_0, a, \sigma_\tau, \epsilon) = (1-\epsilon) G\left(\tau_i - \bar{\tau}([\mathrm{Fe/H}]_i), \sqrt{\sigma_\tau^2 + \delta\tau_i^2}\right) + \tag{27}$$

$$\frac{\epsilon}{2(\tau_{max} - \tau_{min})}\left(\mathrm{erf}(\frac{\tau_{max} - \tau}{\sqrt{2}\delta\tau_i}) - \mathrm{erf}(\frac{\tau_{min} - \tau}{\sqrt{2}\delta\tau_i})\right), \tag{28}$$

and the total log-likelihood function

$$\ln\, P(\{\tau_i, \delta\tau_i, [\mathrm{Fe/H}]_i\} | \bar{\tau}_0, a, \sigma_\tau, \epsilon) = \Sigma \ln\, P_i(\tau_i, \delta\tau_i | [\mathrm{Fe/H}]_i, \bar{\tau}_0, a, \sigma_\tau, \epsilon), \tag{29}$$

For this analysis we selected stars of the old, thick disk with a cut in both momentum and metallicity: $-1.0 < [\mathrm{Fe/H}] < -0.2$. We realized that there could have substantial contamination if we simply had taken all stars with low angular momentum or high $[\alpha/\mathrm{Fe}]$ as thick disk stars. The major contamination source is likely the blue straggler stars (BSSs), which are products of binary evolution [30, 31]. Due to the binary mass transfer (or merger), these stars have larger masses (thus younger ages) than the single stars of the same underlying population. To minimize impact from contaminators and outliers, we therefore divide the low-angular momentum or high-alpha stars into 0.1 dex bin in the [Fe/H] range of [-1.0,-0.2] dex, and determine the mean age and dispersion in each bin with Gaussian fit to the age distribution. We then discard stars with age deviated from the mean age by more than 2σ, and using the remaining stars as the thick disk stars for estimating the intrinsic age scatter. After applying this iteration for removing outliers, we re-fit the model with the outlier parameter ϵ setting to be zero.

Extended Figure 1 shows the results of the MCMC fits to the parameters a, $\bar{\tau}_0$, and σ_τ. The results suggest that the thick disk sequence has an age of about 9.3 Gyr at $[\mathrm{Fe/H}] = 0$. At a constant [Fe/H], the intrinsic age scatter of the thick disk stars is about 0.82 ± 0.01 Gyr. Given the slope of -3.8 Gyr/dex, this means that at a given age, the [Fe/H] dispersion of the thick disk is 0.22 dex. This is a relative small value compared to the thin disk, for which the strong radial [Fe/H] gradient for stars of the same age [$\simeq -0.1$ dex/kpc, e.g. 21] and the mixing effect may lead to larger [Fe/H] dispersion [e.g. 32]. To this extent, it means that the thick disk was formed from well mixed gas. However, a dispersion of 0.22 dex is large compared to the [Fe/H] dispersion of the young stars at a given Galactocentric radius, which is expected to $\lesssim$ 0.1 dex[e.g. 33]. However, we emphasize that since our sample stars of the 'thick' disk may still suffer some contamination from, e.g., the BSSs population, the inner thin disk population, etc., the derived age scatter (and [Fe/H] scatter) of the thick disk is likely to be overestimated. While the estimate of the mean age as a function of [Fe/H] is robust.

4. The old disk stars "splashed" by the merger with Gaia-Sausage-Enceladus

Recent studies have suggested that the Milky Way's inner halo is dominated by stars that were accreted from a satellite galaxy, dubbed Gaia Enceladus [34] or Gaia Sausage [35] (GSE). This merger would have strongly perturbed the orbits of stars in any preexisting in-situ Milky Way disk. Given GSE's low-angular momentum orbit, the pre-existing stars might also have low-angular momentum orbits after the merger: they should appear kinematically like halo stars (close to zero angular momentum) with abundances similar to the oldest in-situ disk ([Fe/H] > -1, high [α/Fe]) [36–39]. This is what appears in the bottom panel of Fig. 3. To follow-up on this we show **Extended Figure 2**, which clearly shows the 'splashed' oldest disk population in the J_ϕ-[Fe/H] plane: its distribution exhibits a sharp drop at an [Fe/H] of -0.4 (see the regime delineated by the red dashed lines).

5. Comparison of age-metallicity relation with literature

The existence of two sequences in stellar age-[Fe/H] distribution of disk stars can be at least traced back to Xiang et al. (2017) [40], who looked into the stellar age-[Fe/H] distribution for nearly a million disk main-sequence turn-off and subgaint stars from LAMOST, and pointed out that the distribution is a superposition of two separate sequences (see their Figure 22 and Sect. 6.4). However, as [40] focussed on presenting the age and mass determination, the age-[Fe/H] relation was only a by-product of their paper and was not investigated in detail. Nissen et al. (2020) [41] presented clear evidence for a bi-modal age-metallicity distribution among disk stars using a small set of stellar ages from high-resolution spectroscopy. This is qualitatively consistent with our data, although the sample of Nissen et al. is restricted at [Fe/H] $\gtrsim -0.3$, while our results suggest that a large portion of the sequences are more metal-poor (**Extended Figure 6**). Interestingly, many stars in the older sequence of Nissen et al. [41] seem to be located in the upper sequence that exhibits positive age-[Fe/H] trend in our results, which means they are probably stars migrated from the inner, thin disk. Given the complex behaviour of old stars at [Fe/H] $\gtrsim -0.2$, the nature of these stars might need to be further studied. Recently, Sahlholdt et al. [42] have studied the age-metallicity relation of Galactic disks using GALAH survey data, and found multi sequences and peaks of the age distribution, suggesting the Galactic disk formation has experienced a multiphase formation. Particularly, they suggested that the age distribution exhibit even more than two peaks. These are qualitatively consistent with what we found here. However, a quantitative comparison would require that one carefully addresses several factors, such as the difference in spatial coverage of the samples, systematics in age determination, different ways of analysing and explaining the data, etc.

In addition, there are several other relevant works. For example, Haywood et al. [43] suggested that the Milky Way is composed of a 'thick' disk, an inner thin disk, and an outer disk, which have different origins. They suggested that

the thick disk was formed from well-mixed gas in a period of 4-5 Gyrs. Snaith et al. [44] estimated the disk star formation history using age–[Si/Fe] relation, and found a dip in star formation rate at ~ 8 Gyr ago. These pictures are qualitatively consistent with what we found in this work, but with a more comprehensive and clear picture due to much larger sample size and spatial coverage, wider metallicity range, as well as more precise age determinations as we focused on subgiant stars only. Bonaca et al. [38] studied the age distribution for old stellar populations in our Galaxy with 11,000 main-sequence turn-off stars from the H3 Survey [45]. They found stars with high [α/Fe] having age older than 8.2 Gyr, which is well consistent with our results, taking into account the fact that minor difference is expected as we use different definition for the 'high-[α/Fe] sequence'. They also found that the metal-poor halo stars have ages truncated at 10.2 Gyr. This is again consistent with our results for the metal-poor ([Fe/H] $\lesssim -1$) sequence in the bottom right panel of Fig. 2.

In **Extended Figure 6**, we have also compared our results with globular clusters (GCs) in the halo and thick disk. The globular cluster sample adopted is a compilation of Forbes et al. [46], VandenBerg et al.[47], and Cohen et al. [48]. For GCs from Forbes et al. [46], only the metal-poor ones with [Fe/H] < -1.2 are adopted, as the more metal-rich ones are found to suffer large systematics by comparing with the VandenBerg et al. [47] for common objects (see Massari et al. [49]). For GCs in both Forbes et al. [46] and VandenBerg et al. [47], we adopt the latter. Note that in **Extended Figure 6** we have normalized the density of our full sample stars for each [Fe/H] , without dividing them into groups of different angular momentum. The prominent sequences shown in Fig. 2 are well presented in **Extended Figure 6** except for that, there is a 'break' in the old disk sequence at [Fe/H] $\simeq -0.2$ and $\tau \simeq 10$ Gyr. This 'break' occurs because young, thin disk stars dominate the populations at [Fe/H] $\simeq -0.2$.

It is remarkable that the distribution of the GCs in the age-[Fe/H] plane for both the halo ([Fe/H] $\lesssim -1$) and the thick disk ([Fe/H] $\gtrsim -1$) are well consistent with our sample stars, except for the very metal-poor end at [Fe/H] < -2, where the mean age of our sample stars is older than that of the GCs by about 1 Gyr. Given the small number and mixed sources of the GC sample, as well as the large age dispersion of our sample stars, the reason for this difference is not clear yet. However, it is no doubt that the halo populations need to be further studied in more detail given that the stars may have complex origins [39, 50].

Taken together, this discussion shows that the hints of the features in the age-[Fe/H] distribution that we have presented here, could be gleamed in a number of earlier works, but never with the sample size or data quality that would have permitted to draw the inferences we have drawn here.

References

[1] Xiang, M., Rix, H.-W., Ting, Y.-S., Zari, E., El-Badry, K., Yuan,

H.-B., Cui, W.-Y.: Data-driven Spectroscopic Estimates of Absolute Magnitude, Distance, and Binarity: Method and Catalog of 16,002 O- and B-type Stars from LAMOST. ApJ Supplement series **253**(1), 22 (2021) arXiv:2008.10637 [astro-ph.SR]. https://doi.org/10.3847/1538-4365/abd6ba

[2] Skrutskie, M.F., Cutri, R.M., Stiening, R., Weinberg, M.D., Schneider, S., Carpenter, J.M., Beichman, C., Capps, R., Chester, T., Elias, J., Huchra, J., Liebert, J., Lonsdale, C., Monet, D.G., Price, S., Seitzer, P., Jarrett, T., Kirkpatrick, J.D., Gizis, J.E., Howard, E., Evans, T., Fowler, J., Fullmer, L., Hurt, R., Light, R., Kopan, E.L., Marsh, K.A., McCallon, H.L., Tam, R., Van Dyk, S., Wheelock, S.: The Two Micron All Sky Survey (2MASS). AJ **131**(2), 1163–1183 (2006). https://doi.org/10.1086/498708

[3] Bailer-Jones, C.A.L., Rybizki, J., Fouesneau, M., Demleitner, M., Andrae, R.: Estimating Distances from Parallaxes. V. Geometric and Photogeometric Distances to 1.47 Billion Stars in Gaia Early Data Release 3. AJ **161**(3), 147 (2021) arXiv:2012.05220 [astro-ph.SR]. https://doi.org/10.3847/1538-3881/abd806

[4] Xiang, M., Ting, Y.-S., Rix, H.-W., Sandford, N., Buder, S., Lind, K., Liu, X.-W., Shi, J.-R., Zhang, H.-W.: Abundance Estimates for 16 Elements in 6 Million Stars from LAMOST DR5 Low-Resolution Spectra. ApJ Supplement series **245**(2), 34 (2019) arXiv:1908.09727 [astro-ph.SR]. https://doi.org/10.3847/1538-4365/ab5364

[5] Lindegren, L., Bastian, U., Biermann, M., Bombrun, A., de Torres, A., Gerlach, E., Geyer, R., Hernández, J., Hilger, T., Hobbs, D., Klioner, S.A., Lammers, U., McMillan, P.J., Ramos-Lerate, M., Steidelmüller, H., Stephenson, C.A., van Leeuwen, F.: Gaia Early Data Release 3. Parallax bias versus magnitude, colour, and position. A&A **649**, 4 (2021) arXiv:2012.01742 [astro-ph.IM]. https://doi.org/10.1051/0004-6361/202039653

[6] Riello, M., De Angeli, F., Evans, D.W., Montegriffo, P., Carrasco, J.M., Busso, G., Palaversa, L., Burgess, P.W., Diener, C., Davidson, M., Rowell, N., Fabricius, C., Jordi, C., Bellazzini, M., Pancino, E., Harrison, D.L., Cacciari, C., van Leeuwen, F., Hambly, N.C., Hodgkin, S.T., Osborne, P.J., Altavilla, G., Barstow, M.A., Brown, A.G.A., Castellani, M., Cowell, S., De Luise, F., Gilmore, G., Giuffrida, G., Hidalgo, S., Holland, G., Marinoni, S., Pagani, C., Piersimoni, A.M., Pulone, L., Ragaini, S., Rainer, M., Richards, P.J., Sanna, N., Walton, N.A., Weiler, M., Yoldas, A.: Gaia Early Data Release 3. Photometric content and validation. A&A **649**, 3 (2021) arXiv:2012.01916 [astro-ph.IM]. https://doi.org/10.1051/0004-6361/202039587

[7] Demarque, P., Woo, J.-H., Kim, Y.-C., Yi, S.K.: Y^2 Isochrones with an Improved Core Overshoot Treatment. ApJ Supplement series **155**(2), 667–674 (2004). https://doi.org/10.1086/424966

[8] Dotter, A., Conroy, C., Cargile, P., Asplund, M.: The Influence of Atomic Diffusion on Stellar Ages and Chemical Tagging. ApJ **840**(2), 99 (2017) arXiv:1704.03465 [astro-ph.SR]. https://doi.org/10.3847/1538-4357/aa6d10

[9] Grevesse, N., Noels, A., Sauval, A.J.: Standard Abundances. In: Holt, S.S., Sonneborn, G. (eds.) Cosmic Abundances. Astronomical Society of the Pacific Conference Series, vol. 99, p. 117 (1996)

[10] Asplund, M., Grevesse, N., Sauval, A.J., Scott, P.: The Chemical Composition of the Sun. ARAA **47**(1), 481–522 (2009) arXiv:0909.0948 [astro-ph.SR]. https://doi.org/10.1146/annurev.astro.46.060407.145222

[11] Carpenter, J.M.: Color Transformations for the 2MASS Second Incremental Data Release. AJ **121**(5), 2851–2871 (2001) arXiv:astro-ph/0101463 [astro-ph]. https://doi.org/10.1086/320383

[12] Xiang, M., Shi, J., Liu, X., Yuan, H., Chen, B., Huang, Y., Wang, C., Wu, Y., Tian, Z., Huo, Z., Zhang, H., Zhang, M.: Stellar Mass Distribution and Star Formation History of the Galactic Disk Revealed by Mono-age Stellar Populations from LAMOST. ApJ Supplement series **237**, 33 (2018) arXiv:1807.04592. https://doi.org/10.3847/1538-4365/aad237

[13] Kroupa, P.: On the variation of the initial mass function. MNRAS **322**, 231–246 (2001) astro-ph/0009005. https://doi.org/10.1046/j.1365-8711.2001.04022.x

[14] Yuan, H., Liu, X., Xiang, M., Huang, Y., Chen, B.: Stellar Loci. I. Metallicity Dependence and Intrinsic Widths. ApJ **799**(2), 134 (2015) arXiv:1412.1232 [astro-ph.SR]. https://doi.org/10.1088/0004-637X/799/2/134

[15] Schlegel, D.J., Finkbeiner, D.P., Davis, M.: Maps of Dust Infrared Emission for Use in Estimation of Reddening and Cosmic Microwave Background Radiation Foregrounds. ApJ **500**, 525–553 (1998) astro-ph/9710327. https://doi.org/10.1086/305772

[16] Schlafly, E.F., Finkbeiner, D.P., Schlegel, D.J., Jurić, M., Ivezić, Ž., Gibson, R.R., Knapp, G.R., Weaver, B.A.: The Blue Tip of the Stellar Locus: Measuring Reddening with the Sloan Digital Sky Survey. **725**(1), 1175–1191 (2010) arXiv:1009.4933 [astro-ph.GA]. https://doi.org/10.1088/0004-637X/725/1/1175

[17] Castelli, F., Kurucz, R.L.: New Grids of ATLAS9 Model Atmospheres. In: Piskunov, N., Weiss, W.W., Gray, D.F. (eds.) Modelling of Stellar Atmospheres. IAU Symposium, vol. 210, p. 20 (2003)

[18] Fitzpatrick, E.L.: Correcting for the Effects of Interstellar Extinction. PASP **111**, 63–75 (1999) astro-ph/9809387. https://doi.org/10.1086/316293

[19] Green, G.M., Schlafly, E., Zucker, C., Speagle, J.S., Finkbeiner, D.: A 3D Dust Map Based on Gaia, Pan-STARRS 1, and 2MASS. ApJ **887**(1), 93 (2019) arXiv:1905.02734 [astro-ph.GA]. https://doi.org/10.3847/1538-4357/ab5362

[20] Bovy, J.: galpy: A python Library for Galactic Dynamics. ApJ Supplement series **216**(2), 29 (2015) arXiv:1412.3451 [astro-ph.GA]. https://doi.org/10.1088/0067-0049/216/2/29

[21] Xiang, M.-S., Liu, X.-W., Yuan, H.-B., Huang, Y., Wang, C., Ren, J.-J., Chen, B.-Q., Sun, N.-C., Zhang, H.-W., Huo, Z.-Y., Rebassa-Mansergas, A.: The evolution of stellar metallicity gradients of the Milky Way disk from LSS-GAC main sequence turn-off stars: a two-phase disk formation history? Research in Astronomy and Astrophysics **15**(8), 1209 (2015) arXiv:1505.08063 [astro-ph.GA]. https://doi.org/10.1088/1674-4527/15/8/009

[22] Luo, A.-L., Zhao, Y.-H., Zhao, G., Deng, L.-C., Liu, X.-W., Jing, Y.-P., Wang, G., Zhang, H.-T., Shi, J.-R., Cui, X.-Q., Chu, Y.-Q., Li, G.-P., Bai, Z.-R., Wu, Y., Cai, Y., Cao, S.-Y., Cao, Z.-H., Carlin, J.L., Chen, H.-Y., Chen, J.-J., Chen, K.-X., Chen, L., Chen, X.-L., Chen, X.-Y., Chen, Y., Christlieb, N., Chu, J.-R., Cui, C.-Z., Dong, Y.-Q., Du, B., Fan, D.-W., Feng, L., Fu, J.-N., Gao, P., Gong, X.-F., Gu, B.-Z., Guo, Y.-X., Han, Z.-W., He, B.-L., Hou, J.-L., Hou, Y.-H., Hou, W., Hu, H.-Z., Hu, N.-S., Hu, Z.-W., Huo, Z.-Y., Jia, L., Jiang, F.-H., Jiang, X., Jiang, Z.-B., Jin, G., Kong, X., Kong, X., Lei, Y.-J., Li, A.-H., Li, C.-H., Li, G.-W., Li, H.-N., Li, J., Li, Q., Li, S., Li, S.-S., Li, X.-N., Li, Y., Li, Y.-B., Li, Y.-P., Liang, Y., Lin, C.-C., Liu, C., Liu, G.-R., Liu, G.-Q., Liu, Z.-G., Lu, W.-Z., Luo, Y., Mao, Y.-D., Newberg, H., Ni, J.-J., Qi, Z.-X., Qi, Y.-J., Shen, S.-Y., Shi, H.-M., Song, J., Song, Y.-H., Su, D.-Q., Su, H.-J., Tang, Z.-H., Tao, Q.-S., Tian, Y., Wang, D., Wang, D.-Q., Wang, F.-F., Wang, G.-M., Wang, H., Wang, H.-C., Wang, J., Wang, J.-N., Wang, J.-L., Wang, J.-P., Wang, J.-X., Wang, L., Wang, M.-X., Wang, S.-G., Wang, S.-Q., Wang, X., Wang, Y.-N., Wang, Y., Wang, Y.-F., Wang, Y.-F., Wei, P., Wei, M.-Z., Wu, H., Wu, K.-F., Wu, X.-B., Wu, Y.-Z., Xing, X.-Z., Xu, L.-Z., Xu, X.-Q., Xu, Y., Yan, T.-S., Yang, D.-H., Yang, H.-F., Yang, H.-Q., Yang, M., Yao, Z.-Q., Yu, Y., Yuan, H., Yuan, H.-B., Yuan, H.-L., Yuan, W.-M., Zhai, C., Zhang, E.-P., Zhang, H.-W., Zhang, J.-N., Zhang,

L.-P., Zhang, W., Zhang, Y., Zhang, Y.-X., Zhang, Z.-C., Zhao, M., Zhou, F., Zhou, X., Zhu, J., Zhu, Y.-T., Zou, S.-C., Zuo, F.: The first data release (DR1) of the LAMOST regular survey. Research in Astronomy and Astrophysics **15**(8), 1095 (2015) arXiv:1505.01570 [astro-ph.GA]. https://doi.org/10.1088/1674-4527/15/8/002

[23] Gravity Collaboration, Abuter, R., Amorim, A., Bauböck, M., Berger, J.P., Bonnet, H., Brandner, W., Clénet, Y., Coudé Du Foresto, V., de Zeeuw, P.T., Dexter, J., Duvert, G., Eckart, A., Eisenhauer, F., Förster Schreiber, N.M., Garcia, P., Gao, F., Gendron, E., Genzel, R., Gerhard, O., Gillessen, S., Habibi, M., Haubois, X., Henning, T., Hippler, S., Horrobin, M., Jiménez-Rosales, A., Jocou, L., Kervella, P., Lacour, S., Lapeyrère, V., Le Bouquin, J.-B., Léna, P., Ott, T., Paumard, T., Perraut, K., Perrin, G., Pfuhl, O., Rabien, S., Rodriguez Coira, G., Rousset, G., Scheithauer, S., Sternberg, A., Straub, O., Straubmeier, C., Sturm, E., Tacconi, L.J., Vincent, F., von Fellenberg, S., Waisberg, I., Widmann, F., Wieprecht, E., Wiezorrek, E., Woillez, J., Yazici, S.: A geometric distance measurement to the Galactic center black hole with 0.3% uncertainty. A&A **625**, 10 (2019) arXiv:1904.05721 [astro-ph.GA]. https://doi.org/10.1051/0004-6361/201935656

[24] Huang, Y., Liu, X.-W., Yuan, H.-B., Xiang, M.-S., Huo, Z.-Y., Chen, B.-Q., Zhang, Y., Hou, Y.-H.: Determination of the local standard of rest using the LSS-GAC DR1. MNRAS **449**(1), 162–174 (2015) arXiv:1501.07095 [astro-ph.GA]. https://doi.org/10.1093/mnras/stv204

[25] Rix, H.-W., Hogg, D.W., Boubert, D., Brown, A.G.A., Casey, A., Drimmel, R., Everall, A., Fouesneau, M., Price-Whelan, A.M.: Selection Functions in Astronomical Data Modeling, with the Space Density of White Dwarfs as a Worked Example. **162**(4), 142 (2021) arXiv:2106.07653 [astro-ph.IM]. https://doi.org/10.3847/1538-3881/ac0c13

[26] Rybizki, J., Demleitner, M., Fouesneau, M., Bailer-Jones, C., Rix, H.-W., Andrae, R.: A Gaia DR2 Mock Stellar Catalog. PASP **130**(989), 074101 (2018) arXiv:1804.01427 [astro-ph.GA]. https://doi.org/10.1088/1538-3873/aabd70

[27] Bressan, A., Marigo, P., Girardi, L., Salasnich, B., Dal Cero, C., Rubele, S., Nanni, A.: PARSEC: stellar tracks and isochrones with the PAdova and TRieste Stellar Evolution Code. MNRAS **427**, 127–145 (2012) arXiv:1208.4498 [astro-ph.SR]. https://doi.org/10.1111/j.1365-2966.2012.21948.x

[28] Nandakumar, G., Schultheis, M., Hayden, M., Rojas-Arriagada, A., Kordopatis, G., Haywood, M.: Effects of the selection function on metallicity trends in spectroscopic surveys of the Milky Way. A&A

606, 97 (2017) arXiv:1707.09932 [astro-ph.GA]. https://doi.org/10.1051/0004-6361/201731099

[29] Chen, B.-Q., Liu, X.-W., Yuan, H.-B., Xiang, M.-S., Huang, Y., Wang, C., Zhang, H.-W., Tian, Z.-J.: The selection function of the LAMOST Spectroscopic Survey of the Galactic Anti-centre. MNRAS **476**(3), 3278–3289 (2018) arXiv:1802.06777 [astro-ph.GA]. https://doi.org/10.1093/mnras/sty454

[30] Chen, X., Han, Z.: Mass transfer from a giant star to a main-sequence companion and its contribution to long-orbital-period blue stragglers. **387**(4), 1416–1430 (2008) arXiv:0804.2294 [astro-ph]. https://doi.org/10.1111/j.1365-2966.2008.13334.x

[31] Chen, X., Han, Z.: Binary coalescence from case A evolution: mergers and blue stragglers. **384**(4), 1263–1276 (2008) arXiv:0710.4601 [astro-ph]. https://doi.org/10.1111/j.1365-2966.2007.12617.x

[32] Frankel, N., Rix, H.-W., Ting, Y.-S., Ness, M., Hogg, D.W.: Measuring Radial Orbit Migration in the Galactic Disk. ApJ **865**(2), 96 (2018) arXiv:1805.09198 [astro-ph.GA]. https://doi.org/10.3847/1538-4357/aadba5

[33] Kreckel, K., Ho, I.-T., Blanc, G.A., Groves, B., Santoro, F., Schinnerer, E., Bigiel, F., Chevance, M., Congiu, E., Emsellem, E., Faesi, C., Glover, S.C.O., Grasha, K., Kruijssen, J.M.D., Lang, P., Leroy, A.K., Meidt, S.E., McElroy, R., Pety, J., Rosolowsky, E., Saito, T., Sandstrom, K., Sanchez-Blazquez, P., Schruba, A.: Mapping Metallicity Variations across Nearby Galaxy Disks. ApJ **887**(1), 80 (2019) arXiv:1910.07190 [astro-ph.GA]. https://doi.org/10.3847/1538-4357/ab5115

[34] Helmi, A., Babusiaux, C., Koppelman, H.H., Massari, D., Veljanoski, J., Brown, A.G.A.: The merger that led to the formation of the Milky Way's inner stellar halo and thick disk. Nature **563**(7729), 85–88 (2018) arXiv:1806.06038 [astro-ph.GA]. https://doi.org/10.1038/s41586-018-0625-x

[35] Belokurov, V., Erkal, D., Evans, N.W., Koposov, S.E., Deason, A.J.: Co-formation of the disc and the stellar halo. MNRAS **478**(1), 611–619 (2018) arXiv:1802.03414 [astro-ph.GA]. https://doi.org/10.1093/mnras/sty982

[36] Belokurov, V., Sanders, J.L., Fattahi, A., Smith, M.C., Deason, A.J., Evans, N.W., Grand, R.J.J.: The biggest splash. MNRAS **494**(3), 3880–3898 (2020) arXiv:1909.04679 [astro-ph.GA]. https://doi.org/10.1093/mnras/staa876

[37] Di Matteo, P., Haywood, M., Lehnert, M.D., Katz, D., Khoperskov, S.,

Snaith, O.N., Gómez, A., Robichon, N.: The Milky Way has no in-situ halo other than the heated thick disc. Composition of the stellar halo and age-dating the last significant merger with Gaia DR2 and APOGEE. **632**, 4 (2019) arXiv:1812.08232 [astro-ph.GA]. https://doi.org/10.1051/0004-6361/201834929

[38] Bonaca, A., Conroy, C., Cargile, P.A., Naidu, R.P., Johnson, B.D., Zaritsky, D., Ting, Y.-S., Caldwell, N., Han, J.J., van Dokkum, P.: Timing the Early Assembly of the Milky Way with the H3 Survey. ApJ Letter **897**(1), 18 (2020) arXiv:2004.11384 [astro-ph.GA]. https://doi.org/10.3847/2041-8213/ab9caa

[39] Helmi, A.: Streams, Substructures, and the Early History of the Milky Way. ARAA **58**, 205–256 (2020) arXiv:2002.04340 [astro-ph.GA]. https://doi.org/10.1146/annurev-astro-032620-021917

[40] Xiang, M., Liu, X., Shi, J., Yuan, H., Huang, Y., Chen, B., Wang, C., Tian, Z., Wu, Y., Yang, Y., Zhang, H., Huo, Z., Ren, J.: The Ages and Masses of a Million Galactic-disk Main-sequence Turnoff and Subgiant Stars from the LAMOST Galactic Spectroscopic Surveys. ApJ Supplement series **232**, 2 (2017) arXiv:1707.06236 [astro-ph.SR]. https://doi.org/10.3847/1538-4365/aa80e4

[41] Nissen, P.E., Christensen-Dalsgaard, J., Mosumgaard, J.R., Silva Aguirre, V., Spitoni, E., Verma, K.: High-precision abundances of elements in solar-type stars. Evidence of two distinct sequences in abundance-age relations. A&A **640**, 81 (2020) arXiv:2006.06013 [astro-ph.SR]. https://doi.org/10.1051/0004-6361/202038300

[42] Sahlholdt, C.L., Feltzing, S., Feuillet, D.K.: Characterizing epochs of star formation across the Milky Way disc using age-metallicity distributions of GALAH stars. (2021) arXiv:2112.08218 [astro-ph.GA]. https://doi.org/10.1093/mnras/stab3681

[43] Haywood, M., Di Matteo, P., Lehnert, M.D., Katz, D., Gómez, A.: The age structure of stellar populations in the solar vicinity. Clues of a two-phase formation history of the Milky Way disk. A&A **560**, 109 (2013) arXiv:1305.4663 [astro-ph.GA]. https://doi.org/10.1051/0004-6361/201321397

[44] Snaith, O.N., Haywood, M., Di Matteo, P., Lehnert, M.D., Combes, F., Katz, D., Gómez, A.: The Dominant Epoch of Star Formation in the Milky Way Formed the Thick Disk. **781**(2), 31 (2014) arXiv:1401.1835 [astro-ph.GA]. https://doi.org/10.1088/2041-8205/781/2/L31

[45] Conroy, C., Bonaca, A., Cargile, P., Johnson, B.D., Caldwell, N.,

Naidu, R.P., Zaritsky, D., Fabricant, D., Moran, S., Rhee, J., Szentgyorgyi, A., Berlind, P., Calkins, M.L., Kattner, S., Ly, C.: Mapping the Stellar Halo with the H3 Spectroscopic Survey. ApJ **883**(1), 107 (2019) arXiv:1907.07684 [astro-ph.GA]. https://doi.org/10.3847/1538-4357/ab38b8

[46] Forbes, D.A., Bridges, T.: Accreted versus in situ Milky Way globular clusters. MNRAS **404**(3), 1203–1214 (2010) arXiv:1001.4289 [astro-ph.GA]. https://doi.org/10.1111/j.1365-2966.2010.16373.x

[47] VandenBerg, D.A., Brogaard, K., Leaman, R., Casagrande, L.: The Ages of 55 Globular Clusters as Determined Using an Improved $\hat{V}$HB_TO Method along with Color-Magnitude Diagram Constraints, and Their Implications for Broader Issues. ApJ **775**(2), 134 (2013) arXiv:1308.2257 [astro-ph.GA]. https://doi.org/10.1088/0004-637X/775/2/134

[48] Cohen, R.E., Bellini, A., Casagrande, L., Brown, T.M., Correnti, M., Kalirai, J.S.: Relative Ages of Nine Inner Milky Way Globular Clusters from Proper Motion Cleaned Color-Magnitude Diagrams. arXiv e-prints, 2109–08708 (2021) arXiv:2109.08708 [astro-ph.GA]

[49] Massari, D., Koppelman, H.H., Helmi, A.: Origin of the system of globular clusters in the Milky Way. A&A **630**, 4 (2019) arXiv:1906.08271 [astro-ph.GA]. https://doi.org/10.1051/0004-6361/201936135

[50] Naidu, R.P., Conroy, C., Bonaca, A., Johnson, B.D., Ting, Y.-S., Caldwell, N., Zaritsky, D., Cargile, P.A.: Evidence from the H3 Survey That the Stellar Halo Is Entirely Comprised of Substructure. **901**(1), 48 (2020) arXiv:2006.08625 [astro-ph.GA]. https://doi.org/10.3847/1538-4357/abaef4
